# Prevalence and socioeconomic factors of diabetes: a population-based cross-sectional analysis from Jordan

**DOI:** 10.7189/jogh.15.04095

**Published:** 2025-03-14

**Authors:** Ghaith M Al-Taani, Austen El-Osta, Saja A Alnahar

**Affiliations:** 1 Department of Clinical Pharmacy and Pharmacy Practice, Faculty of Pharmacy, Yarmouk University, Irbid, Jordan; 2 Self-Care Academic Research Unit (SCARU), Imperial College London Department of Primary Care & Public Health, London, UK; 3 Institute of Public Health, The University of Jordan, Amman, Jordan

## Abstract

**Background:**

Diabetes mellitus (DM) is a significant public health issue in Jordan. While several studies have investigated the biological and behavioural determinants' influence on diabetes mellitus incidence, prevalence and outcomes, few studies investigated socioeconomic factors. Moreover, the available Jordan-based literature lacks an investigation of the influence of socioeconomic factors on diabetes outcomes. This research seeks to evaluate the incidence of DM among the adult population of Jordan and examine the influence of significant socioeconomic variables.

**Methods:**

The research team used the 2017–2018 Jordanian Demographic and Health Survey of individuals aged 40 and older. We used self-reported data to assess the prevalence and onset of diabetes. Descriptive and inferential analyses assessed the association between diabetes and the extracted socioeconomic variables.

**Results:**

Of the 21 860 extracted records, 3443 (15.8%) were related to diabetic patients. Approximately, 60% of those who were diagnosed with DM were in the 40–59 years age range. Statistically significant associations were found between the prevalence of DM and socioeconomic factors. Older age and low educational attainment were significantly associated with a higher prevalence of DM. The wealth index and residential location were also significantly associated with DM prevalence.

**Conclusion:**

The study findings emphasise the need for specifically tailored and delivered public health interventions targeting individuals with challenging socioeconomic factors such as older age and low educational attainment. Healthcare providers and policymakers should focus on delivering and sponsoring educational and awareness programmes to promote self-care and the adoption of health-seeking lifestyle behaviours and practices among unprivileged and at-risk local communities. Healthcare authorities could initiate community-based diabetes screening programmes targeting marginalised and at-risk groups.

Diabetes mellitus (DM) is a chronic metabolic progressive disease characterised by hyperglycaemia resulting from defects in insulin secretion, insulin resistance or both. Chronic hyperglycaemia, resulting from the disease, culminates into both microvascular and macrovascular complications, including retinopathy, nephropathy, neuropathy, coronary heart disease, peripheral vascular disease and cerebrovascular disease [1]. There are several types of DM; the most frequently reported are DM-type 2, DM-type 1 and gestational diabetes in that same order [2].

Due to its resource-intensive nature, DM represents a significant financial and organisational challenges to global health care systems. The recent decades have witnessed an escalation in DM incidents rates, particularly in low- and middle-income countries (LMICs) [3]. According to the World Health Organization (WHO), DM-type 2 is the most prevalent type of diabetes [3]. The exact aetiology of DM-type 2 has yet to be fully understood. However, it is believed to result from genetic-environment interactions [4], where inadequate lifestyle and behavioural factors such as overweight, poor nutrition, the consumption of sugar-dense foods and low exercise level are superimposed on a susceptible genotype [4].

Socioeconomic factors such as education, income and type of residential area are frequently reported to have a significant effect on the onset and outcomes of lifestyle diseases including DM, hypertension and heart diseases [5]. In the case of DM, many researchers found an association between age, wealth index, educational level and smoking habits [6–10]. While available evidence confirms the association between socioeconomic factors and the onset and prognoses of DM, it also shows contradictions between developed and developing nations. For instance, in 2012, Sacerdote et al. found that poor clinical outcomes of DM were associated with low educational level among the European population [11]. On the other hand, Seiglie et al. found that in LMICs, a higher incidence of DM was linked to a higher educational level [12]. Similar to educational level, income and wealth index were associated with the incidence and outcome of DM; while British citizens with low income and limited financial resources were at risk of developing DM, wealthy Bangladeshis were more susceptible to developing DM. They had poor clinical outcomes and disease prognoses [13,14].

Similar to other LMICs, Jordan has witnessed an increase in DM-type 2 prevalence, which has become a major public health concern with significant financial impacts [15]. In their trend analysis, Ajlouni et al. showed that over twenty years, DM prevalence went from 13.0% in 1997 to 23.7% in 2017 [16]. More recently, Awad et al. estimated that by 2050, Jordan will have more than 1.9 million cases of DM and 62.2% of the population will suffer from obesity [17]. Accordingly, 25% of health expenditure is expected to be directed and dedicated to treating DM patients and helping obese individuals [17]. The estimated high prevalence of DM is attributed to the ageing population, widespread of unhealthy lifestyle behaviours, low physical activities and obesity [18].

The Jordanian health care system is expected to experience an increase in the demand for DM-related services necessitating a need to assess and understand DM patients, patients at risk and the general population's knowledge, attitude and behaviour toward DM. In a recent population-based cross-sectional study, the Jordanian population showed a good understanding and knowledge of DM, its nature, risk factors and complications. However, the study showed that work still needs to be done to improve and enhance the population's attitudes and behaviours toward DM [19].

Tailoring effective and efficient awareness and educational initiatives and programmes requires a deep understanding of the socioeconomic characteristics of DM or at-risk patients. Understanding socioeconomic factors could help find an approach to ameliorate amendable DM-type 2 risk factors.

While there is a substantial amount of literature examining the clinical outcomes and disease prognoses of DM in Jordan, there is a need for population-based studies that establish a connection between DM and the socioeconomic determinants and patients’ characteristics. The aim of this study was to examine the socioeconomic attributes of Jordanian individuals diagnosed with DM and determine how these parameters are linked to the diagnosis of DM. By carrying out a holistic assessment of socioeconomic factors influencing the onset and prognosis of DM using a nationwide data set, this research might be able to identify at-risk and vulnerable individuals. The findings of this study could be utilised to build a comprehensive national policy or framework for health care resource distribution that is essential for identifying individuals at risk and implementing tailored behavioural and social interventions to manage DM effectively, including by promoting self-care approaches [20] that can help prevent, delay the appearance of, or change the trajectory of this debilitating noncommunicable disease (NCD).

## METHODS

### Overall study design and data source

This was a cross-sectional study based on previously collected data. We obtained the data from the 2017–2018 Jordanian Demographic and Health Surveys (DHS). The DHS is a United States Agency for International Development (USAID)-funded programme that aims to collect and summarise data related to the population, health status and health systems of countries around the world [21]. The Jordanian DHS includes nationally collected data on fertility, family planning, child health, gender, lifestyle diseases and nutrition [22]. The data set is freely available to the public and contains no personal identifiers.

### Extracted data

The data used for this study were related to adults aged 40 years or over who reported being diagnosed with DM. Extracted data included patients' sex, age at the time of data collection, marital status, number of household members (family size), place of residence, nationality, educational level, wealth index and income status. We sought permission to access the data from USAID DHS programme in the form of Statistical Package for Social Sciences files.

### Data analysis

We carried out data analysis using descriptive and inferential analysis. Only completed records were considered for analysis.

#### Descriptive analysis

We performed descriptive analysis to provide an overall characterisation of the study population. Data were reported in frequencies and percentages and results were represented using summary tabulation.

#### Inferential analysis

We used inferential analysis to examine and investigate the association between DM diagnosis (the dependent variable) and socioeconomic factors (the independent variables). Inferential analysis was implemented in two steps. Step one: χ^2^ analysis, which identified independent variables associated with DM diagnosis. Only variables with a *P*-value less than 0.250 were considered for the next step. A *P*-value threshold of 0.250 was established to prevent the early exclusion of variables having marginal associations from further analysis in the logistic regression model. This method is often used to enhance the inclusiveness of variables in exploratory studies aimed at identifying possible predictors. In Step two, the backward logistic regression model retained only statistically significant independent variables with the outcome variable. The backward logistic regression was considered to suit the exploratory nature of this study as it allowed for considering all candidate variables and methodically eliminating non-significant variables according to statistical criteria [23,24]. Accordingly, we used only the most relevant predictors in the final model. This approach was favoured over forward selection, which may neglect significant variables omitted early in the procedure and stepwise selection, which may encounter multicollinearity problems [24,25]. Statistical Package for Social Sciences was set to calculate the predicted probability of being a DM patient for every participant by using the assigned weights of variables in the final logistic regression model and the participant's characteristics. The significance level of the present study was set at a *P*-value equal to or less than 0.05.

### Ethical approval

Ethical approvals and reviews for primary data collection were secured by the DHS Programme from the relevant national ethical review boards. As this study sought to use anonymised data acquired with permission from the DHS Programme and did not include direct engagement with human subjects, it did not require further ethical approvals. We handled and performed secondary data by national and international guidelines and standards. We reported this study in compliance with the Strengthening the Reporting of Observational Studies in Epidemiology (STROBE) guidelines.

## RESULTS

### Diabetes mellitus prevalence

From a comprehensive data set of 21 860 records, the team identified 3443 (15.75%) diabetic patients. This accounts for a crude prevalence of DM in 2017–2018 at 157.5 per 1000 population. We identified a significant gender disparity, with a higher prevalence in females at 162.4 per 1000 compared to males at 152.3 per 1000 (*P* = 0.04). Out of the 3443 diabetic patients, 466 (13.5%) reported an early onset of DM, being diagnosed before the age of 40. Further analysis highlighted a correlation between older age and the likelihood of DM (**Table 1**).

**Table 1:** Baseline characteristics of diabetic and non-diabetic individuals

Investigated attributes	Investigated group		
	**Non-diabetic population, n (%)**	**Diabetic population, n (%)**	**Study population, n (%)**
**Demographics and characteristics**			
Gender			
*Male*	9054 (49.2)	1627 (47.3)	10 681 (48.9)
*Female*	9363 (50.8)	1816 (52.7)	11 179 (51.1)
Age group (in years)			
*40–44*	4875 (26.5)	229 (6.7)	5104 (23.3)
*45–49*	4410 (23.9)	404 (11.7)	4814 (22.0)
*50–54*	3164 (17.2)	539 (15.7)	3703 (16.9)
*55–59*	2104 (11.4)	625 (18.2)	2729 (12.5)
*60–64*	1380 (7.5)	517 (15.0)	1897 (8.7)
*65–69*	935 (5.1)	450 (13.1)	1385 (6.3)
*70 y and older*	1549 (8.4)	679 (19.7)	2228 (10.2)
Marital status			
*Never been married*	939 (5.1)	55 (1.6)	994 (4.5)
*Married*	15 583 (84.6)	2691 (78.2)	18 274 (83.6)
*Divorced*	253 (1.4)	37 (1.1)	290 (1.3)
*Separated*	28 (0.2)	6 (0.2)	34 (0.2)
*Widowed*	1614 (8.8)	654 (19.0)	2268 (10.4)
Level of Education			
*Illiterate or no formal education*	2706 (14.7)	843 (24.5)	3549 (16.2)
*Primary education*	2344 (12.7)	626 (18.2)	2970 (13.6)
*Secondary education*	9139 (49.6)	1383 (40.2)	10 522 (48.1)
*University education*	4224 (22.9)	590 (17.1)	4814 (22.0)
*Not reported*	4 (0.02)	1 (0.0)	5 (0.0)
Nationality			
*Jordanian*	16 466 (89.4)	3142 (91.3)	19 608 (89.7)
*Arabic nationalities*	1897 (10.3)	299 (8.7)	2196 (10)
*Other nationalities*	53 (0.3)	2 (0.1)	55 (0.3)
*Not reported*	1 (0.01)	0 (0.0)	1 (0.0)
**Living arrangements**			
Region			
*Northern region*	6110 (33.2)	1244 (36.1)	7354 (33.6)
*Central region*	7218 (39.2)	1438 (41.8)	8656 (39.6)
*Southern region*	5089 (27.6)	761 (22.1)	5850 (26.8)
Wealth index			
*Poorer*	4815 (26.1)	842 (24.5)	5657 (25.9)
*Poor*	4192 (22.8)	793 (23.0)	4985 (22.8)
*Middle*	3738 (20.3)	717 (20.8)	4455 (20.4)
*Rich*	3141 (17.1)	583 (16.9)	3724 (17.0)
*Richer*	2531 (13.7)	508 (14.8)	3039 (13.9)
Number of family members (household size)			
*1–3 members (small)*	3801 (20.6)	1152 (33.5)	4953 (22.7)
*4–9 members (medium)*	13 826 (75.1)	2176 (63.2)	16 002 (73.2)
*10 or more members (large)*	790 (4.3)	115 (3.3)	905 (4.1)
**Health status**			
Age of DM diagnosed (in years)			
*Younger than 20 y*	Not applicable	3 (0.1)	Not applicable
*20–29 y*	Not applicable	59 (1.7)	Not applicable
*30–39 y*	Not applicable	404 (11.8)	Not applicable
*40–49 y*	Not applicable	1119 (32.6)	Not applicable
*50–59 y*	Not applicable	1090 (31.8)	Not applicable
*60 y or older*	Not applicable	754 (22.0)	Not applicable
*Not reported*	Not applicable	14 (0.4)	Not applicable
Years since the first diagnosis (years living with DM)			
*0–4 y*	Not applicable	1166 (5.3)	Not applicable
*5–9 y*	Not applicable	884 (4.0)	Not applicable
*10–14 y*	Not applicable	687 (3.1)	Not applicable
*15–19 y*	Not applicable	331 (1.5)	Not applicable
*20 y or more*	Not applicable	362 (1.7)	Not applicable
*Not reported*	Not applicable	13 (0.4)	Not applicable

DM – diabetes mellitus, N – number

### Socioeconomic and demographic characteristics

Baseline characteristics showed that the percentage of diabetic patients involved in an active relationship was significantly lower than that of non-diabetic patients, 78.2 *vs*. 84.6%; respectively. The two groups also had significantly different household and living arrangements, as the number of diabetic patients who belong to medium to large families was significantly less than that of non-diabetic individuals; those with small family size (1–3 members) were more likely to have diabetes. Moreover, the geographical distribution of DM cases showed that DM patients were more likely to reside in the central and northern regions.

In terms of education, the analysis showed that non-diabetic individuals were significantly more educated than the diabetic population, 85.3 *vs*. 75.5%, respectively. Lastly, DM was more prevalent amongst the Jordanian and Arabic nationalities, excluding Egyptians, Syrians and Iraqis. **Table 1** details the baseline characteristics of diabetic and non-diabetic individuals.

### Socioeconomic factors associated with diabetes

**Figure 1:**
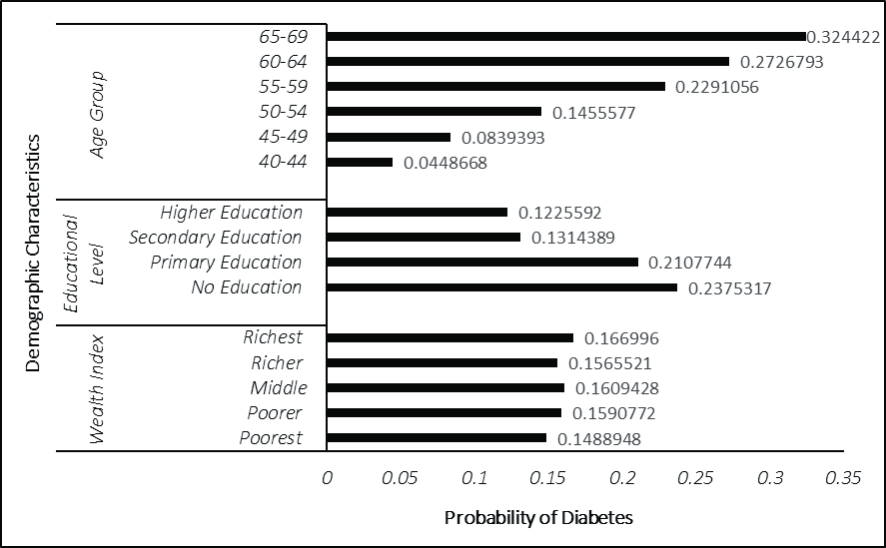
Influence of wealth index, educational level, and age of probability of self-reported diabetes mellites.

The logistic regression analysis revealed that gender, age, level of education, place of residence, family size and wealth index were significantly associated with DM diagnosis (**Table 2**). Further analysis showed that the probability of being diagnosed with DM tends to change in response to wealth index changes. Moreover, DM cases increase with age, as the older population tends to have a significantly higher prevalence of DM. On the other hand, higher education resulted in a lower prevalence of DM (**Figure 1**).

**Table 2:** Association of socioeconomic factors with DM

Category		B (SE)	OR (SE)	(95% CI)
Age, in years				
*40–44*		Reference category		
45–49*		0.658 (0.086)	1.931 (0.086)	1.633–2.283
50–54*		1.273 (0.083)	3.573 (0.083)	3.039–4.201
55–59*		1.802 (0.083)	6.064 (0.083)	5.156–7.131
60–64*		2 (0.088)	7.388 (0.088)	6.221–8.774
65–69*		2.238 (0.093)	9.378 (0.093)	7.817–11.252
70 and older*		2.11 (0.091)	8.249 (0.091)	6.905–9.855
Region				
*Southern*		Reference category		
Central*		0.241 (0.051)	1.273 (0.051)	1.151–1.408
Northern*		0.365 (0.052	1.44 (0.052)	1.301–1.595
Wealth index				
*Poorest*		Reference category		
*Poorer*†		0.161 (0.057)	1.174 (0.057)	1.05–1.314
Middle*		0.256 (0.061)	1.292 (0.061)	1.147–1.455
Richer*		0.273 (0.066)	1.315 (0.066)	1.155–1.496
Richest*		0.3 (0.073)	1.35 (0.073)	1.169–1.558
Family size				
*10 and more*†		Reference category		
*1–3*‡		0.223 (0.111)	1.25 (0.111)	1.005–1.554
*4–9*		0.071 (0.107)	1.073 (0.107)	0.871–1.323
Educational level				
*No education*		Reference category		
*Primary*†		0.197 (0.065)	1.218 (0.065)	1.071–1.384
*Secondary*		−0.049 (0.062)	0.952 (0.062)	0.844–1.075
Higher*		−0.278 (0.076)	0.758 (0.076)	0.653–0.879
Gender				
*Female*‡		0.095 (0.041)	1.1 (0.041)	1.015–1.192
Constant		−3.528 (0.14)	0.029 (0.14)	

B – coefficient, CI – confidence interval, DM – diabetes mellitus, OR – odds ratio, P – probability value, SE – standard error

*P < 0.001.

†*P* = 0.003–0.005.

‡*P* = 0.02–0.05.

## DISCUSSION

The present study evaluated DM prevalence and associated socioeconomic factors among Jordanian adults aged 40 and older, offering practical implications for public health policies and programmes. The emerging evidence informs, empowers and provides a roadmap for targeting DM patients and at-risk individuals and motivating action towards risk factor modification.

This study showed that the crude DM prevalence in Jordanians aged 40-year or over was approximately equal to that of the Middle East and North Africa Region (MENA) region and higher than the global prevalence at 15.8, 16.2, and 10.5% respectively [26]. On the other hand, unlike the global trend, the prevalence of DM among female patients was comparatively higher than among male patients and considerably higher than the global average [26]. Females' higher risk of DM could be attributed to the fact that obesity and overweight – both recognised DM risk factors – are significantly higher among females in the MENA region compared to males [27]. Pertinently, in their paper, Awad et al. predicted that in 2016 the prevalence rate of type-2 DM was 16% [17]; therefore, it is expected that the assessed prevalence of DM in this study might be less than the actual prevalence. The data collection approach which included self-reported diagnosis of DM could also explain the inaccuracy, as some patients might not be aware of their diabetic status, being undiagnosed or not reporting their DM status due to social stigma.

Similar to studies from other countries, DM rates differed based on age, with the highest prevalence and incidence observed in the elderly population [28–30]. Moreover, diabetic elderly patients are more susceptible to complications and mortality than younger ones [31–34]. The current study's results suggest that both the incidence and prevalence of diabetes rise considerably as individuals get older. Comprehending these disparities in age is essential for formulating specific measures to prevent and control diabetes, particularly among older individuals.

While biological and physiological indicators, such as fasting blood glucose and haemoglobin A1C (HbA1c) and physical signs and symptoms, such as frequent urination, thirst and fatigue, are frequently used to diagnose diabetes and assess its prognoses [35], social determinants of health (SDoH), including socioeconomic status (SES), are being perceived as predictors of patients at risk of developing DM and its overall prognoses and diseases outcomes [36,37]. Therefore, identifying SES factors, mainly the level of education, income, employment status, household size and living arrangements of a targeted population, could be used to tailor health care interventions and programmes to enhance disease outcomes and health service quality, equity and accessibility. In general, this study's findings revealed a significant correlation between socioeconomic variables and the incidence of diabetes in Jordan. A higher wealth index has been identified as a significant risk factor for diabetes mellitus, with persons in the wealthiest quintile exhibiting increased chances of diagnosis relative to those in lower wealth categories. Conversely, elevated educational attainment had a protective effect, with individuals with a university degree being less likely to report diabetes mellitus than those with lesser educational qualifications. The results underscore the intricate relationship between socioeconomic status and health behaviours, whereby affluence may enhance access to detrimental lifestyle options. At the same time, education equips people with the information to pursue better practices. These associations were similar to findings from developed and developing countries with different cultural and societal norms and lifestyles [36,38,39].

This study's emerging evidence showed that, similar to other low and middle-income countries, DM prevalence is influenced by educational level, as it tends to be higher among Jordanians with lower educational attainment [12]. This finding could explain why health policy scholars consider the educational level a cornerstone in determining overall individual and population health and well-being [40,41]. Available evidence showed that, in general, a higher educational level might result in higher income and improved access to health care facilities and services, translating to a healthier lifestyle, improved disease outcomes and enhanced quality of life [40,41]. Moreover, diabetes-related assessments showed that individuals with higher levels of education typically possess greater knowledge regarding healthy dietary choices and physical activity, diminishing their susceptibility to diabetes [12,42]. On the other hand, those with lesser education levels may have an inadequate understanding of healthy behaviours, elevating their susceptibility to illness development [12,42].

Logistic regression confirmed a significant association between the wealth index and DM prevalence. However, evidence showed that DM prevalence fluctuated with the wealth index and was not constant. Available literature frequently emphasises the influence of wealth index and income status on DM's incidence, prevalence rate and outcomes [14,36,43,44]. Nevertheless, similar to this study's findings, the influence is multi-dimensional, contradictory and complicated [45]. While several studies showed that in high-income countries, disadvantaged individuals with challenging financial status were more susceptible to being diagnosed with DM [46–49], others showed that wealthy and privileged individuals are at higher risk of developing DM in low and middle-income countries [12,50,51].

Lastly, other socioeconomic factors, such as region of residence and household size, were found to influence DM incidence and prevalence rates. However, despite abundant literature and reports discussing the influence of family history, age, income and education on the prevalence and outcomes of DM [52–54], the literature falls short in investigating the association and influence of household size and the place of residence. However, the observed influence of household size might be attributed to the notion that household size and bigger families are usually struggling financially, especially in LMICs [55,56]. Accordingly, household size might indirectly impact the risk and management of diabetes. Therefore, health policymakers need to tailor interventions and programmes to address and overcome struggles large families face that might affect their health and overall well-being.

The region of residence also influenced the prevalence of DM. Unfortunately, the retrieved data did not include information regarding the specific characteristics of the residential area, such as whether it is classified as rural or urban. The absence of data indicates a possible subject for future investigation, as comprehending the distinct attributes of various living locations could yield significant knowledge regarding DM incidence and prevalence and availability of related health services and programmes.

As DM and its outcomes poses a threat to the general public's health and puts strain on the limited health care systems and resources, it is important to identify and investigate biological and social risk factors. Building on the available literature related to biological risk factors, this study was able to provide an insight into socioeconomic factors that might influence DM incidence, prevalence and outcomes.

This research offers significant insights into the socioeconomic determinants of diabetes prevalence in Jordan; nonetheless, it is crucial to recognise possible unmeasured confounding variables that may have affected the observed relationships. Key lifestyle determinants, such as nutritional practices and level of physical activity, are recognised to influence the risk of diabetes and its outcomes significantly [57]. However, due to limitations related to the extracted data, it was not possible to explore these factors and their associations with socioeconomic factors. Available literature showed that individuals from challenging socioeconomic backgrounds may have restricted access to nutritional food and chances for physical activity, thereby increasing the risk of diabetes development [58,59]. This research examined several socioeconomic aspects; however, significant variables like employment and health care access were excluded due to data constraints. Subsequent studies integrating these characteristics may provide a more thorough understanding of the social and behavioural drivers of diabetes.

### Practical implications of the findings

This study's results emphasise the necessity for focused public health campaigns and interventions within the community to tackle the high prevalence rate of DM, especially among females and senior citizens in Jordan. This includes health literacy and self-care. Accordingly, public health experts and advocates should consider designing and delivering programmes specific to these two demographic groups. Moreover, it is essential to customise community-based programmes and services that address the specific needs of socioeconomically disadvantaged and challenged people. These interventions should prioritise healthy lifestyle behaviours and proper and efficient self-care practices.

Jordanian health policymakers should prioritise the fair allocation of health care services, especially in local communities struggling with high DM prevalence and challenging socioeconomic status. Policymakers should work on creating comprehensive care models that tackle the medical and social needs of DM patients or individuals at risk of developing DM to improve disease management, outcomes and quality of life. Moreover, policymakers should work closely with public health scholars and researchers to prioritise research identifying and investigating the direct impact of place of residence and household size on the incidence of DM and the accessibility to its related health care services and facilities. The findings of these investigations should feed into the development of health care programmes and interventions capable of addressing the challenges related to living arrangements, such as geographical region, household size and marital status.

According to the study's results, specialised interventions should be designed to meet the needs of the at-risk population, mainly women and the elderly. Community-oriented diabetes screening initiatives should be established to promote early detection and treatment of diabetes mellitus, especially in underprivileged and marginalised areas. Moreover, workshops and health awareness campaigns advocating for a healthy lifestyle in terms of a balanced diet, active physical life and reduced stress must be customised for these demographic groups. For women, mitigating obstacles to exercise and enhancing access to health education materials might be especially beneficial. Public health policies must include engagement with local organisations to guarantee cultural and regional relevance, enhancing the reach and efficacy of these programmes.

Policymakers should incorporate health-related subjects into formal academic curricula at different levels, along with primary, secondary and higher education institutions and authorities. The educational programmes should equip individuals with the necessary knowledge and resources to make healthier choices and behaviours. This could be achieved by initiating and establishing a partnership with the Ministry of Education to develop age-appropriate health education modules addressing issues such as nutrition, physical exercise and diabetes prevention. These modules may be integrated into current curricula, such as science or physical education, or presented as independent topics. Public-private collaborations between health care organisations and non-governmental organisations might provide educators with vital resources, training and culturally appropriate materials. Pilot projects may be launched at specific schools to assess the efficacy of these activities and enhance the strategy before countrywide execution. Equipping the general population with information and tools to make better choices and adopt sustainable behaviours might significantly reduce the prevalence of diabetes and other lifestyle-related disorders in Jordan.

Socioeconomic support programmes are essential to tackle income inequality and enhance living conditions. To effectively manage and prevent diabetes in the Jordanian population, health authorities and policymakers should focus on tackling the practical and policy-related consequences associated with this disease.

### Strengths and limitations

A major strength of this study lies in the use of data from DHS, a reputable source of population health information in developing nations. The sample size was also robust, providing a representative profile of adults in Jordan. Given the limited research on the frequency of socioeconomic risk factors for DM in Jordan and other Middle Eastern countries, the study's findings offer valuable insights for health academics and professionals involved in health policy, with the potential to inform future research and policy development in the region. Nevertheless, it is essential to use caution when interpreting the findings due to the reliance on self-reported disease status, which needed to be corroborated by objective measurements. The principal limitation of this study was related to how data on DM was recorded. It is well established that self-reporting may lead to underreporting, particularly in cases of undiagnosed diabetes or where social stigma influences disclosure. As a result, the true prevalence of diabetes may be underestimated, potentially skewing the relationship between diabetes and socioeconomic determinants. Additionally, the cross-sectional nature of the study restricts the ability to establish causal relationships between variables, whereas the absence of an investigation into potential interaction effects among variables in the logistic regression analysis further limits the depth of the findings. Finally, we acknowledge that the generalisability of the results may be constrained by the study's focus on individuals aged 40 and older, which may not fully reflect the broader population.

## CONCLUSIONS

The study revealed significant associations between the diagnosis of diabetes mellitus (DM) and various socioeconomic factors, alongside an assessment of prevalence. A clear link was identified between DM status and the examined determinants, highlighting the need for targeted programmes and interventions that address specific demographic and socioeconomic factors, including age, geographic location, literacy levels and income. These interventions must focus on the unique needs of underprivileged and underserved communities in the country.

Future research should explore the role of residential location and household size in influencing diabetes prevalence, with particular attention to health care accessibility and lifestyle factors. Additionally, incorporating biological markers, such as HbA1c, insulin resistance and fasting blood glucose levels, in conjunction with socioeconomic data, would offer a more comprehensive understanding of diabetes risk and outcomes. Longitudinal studies and mixed-method approaches would be invaluable in further elucidating the complex interactions among these determinants.
